# Extracting Rectified Building Footprints from Traditional Orthophotos: A New Workflow

**DOI:** 10.3390/s22010207

**Published:** 2021-12-29

**Authors:** Qi Chen, Yuanyi Zhang, Xinyuan Li, Pengjie Tao

**Affiliations:** 1 School of Geography and Information Engineering, China University of Geosciences (Wuhan), Wuhan 430074, China; zhangyy@cug.edu.cn (Y.Z.); li.xinyuan@cug.edu.cn (X.L.); 2 School of Remote Sensing and Information Engineering, Wuhan University, Wuhan 430072, China; pjtao@whu.edu.cn

**Keywords:** image segmentation, building footprint, aerial orthophoto, relief displacement

## Abstract

Deep learning techniques such as convolutional neural networks have largely improved the performance of building segmentation from remote sensing images. However, the images for building segmentation are often in the form of traditional orthophotos, where the relief displacement would cause non-negligible misalignment between the roof outline and the footprint of a building; such misalignment poses considerable challenges for extracting accurate building footprints, especially for high-rise buildings. Aiming at alleviating this problem, a new workflow is proposed for generating rectified building footprints from traditional orthophotos. We first use the facade labels, which are prepared efficiently at low cost, along with the roof labels to train a semantic segmentation network. Then, the well-trained network, which employs the state-of-the-art version of EfficientNet as backbone, extracts the roof segments and the facade segments of buildings from the input image. Finally, after clustering the classified pixels into instance-level building objects and tracing out the roof outlines, an energy function is proposed to drive the roof outline to maximally align with the building footprint; thus, the rectified footprints can be generated. The experiments on the aerial orthophotos covering a high-density residential area in Shanghai demonstrate that the proposed workflow can generate obviously more accurate building footprints than the baseline methods, especially for high-rise buildings.

## 1. Introduction

**Figure 1:**
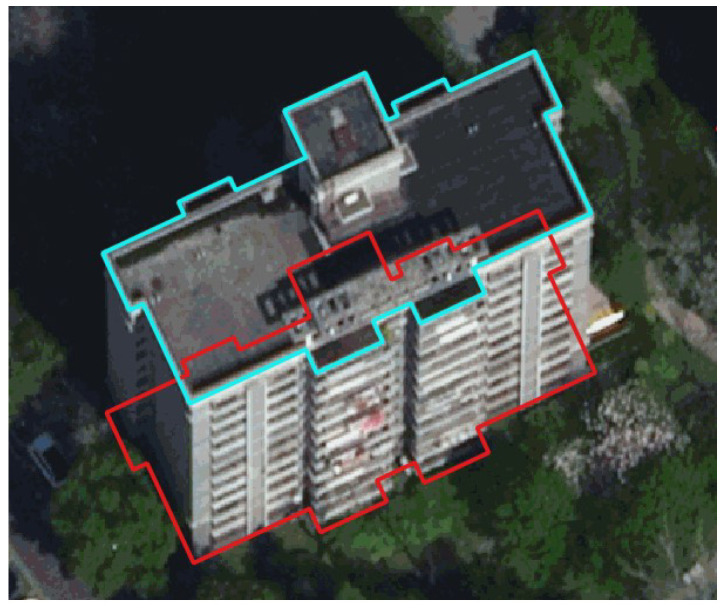
The misalignment between the roof outline (cyan) and the footprint (red) of a building in a traditional orthophoto.

High precision building footprints are one of the most important elements within the geographic vector map of cities, which plays a significant role in many fields, such as urban planning, post-disaster management, carbon emission calculation, and location-based services. The successful application of deep learning techniques such as convolutional neural networks (CNNs) has now greatly improved the accuracy of automatic building detection from remote sensing images [1,2,3]. Despite this achievement, few studies have been proven capable of accurately extracting building footprints (i.e., the boundaries where the building facades meet the ground) from the traditional orthophotos; instead, several previous works focus on segmenting roof surfaces from the input image [4,5,6]. However, for the traditional orthophotos, the roof outline cannot always accurately represent the 2D geographic location of the building footprint. For example, Figure 1 shows a building in an orthophoto which has been properly projected on a digital terrain model (DTM), in which the footprint and the roof outline are largely misaligned due to the tilt effect of the building.

The tilt effect is usually more significant for high-rise buildings; thus, the typical workflow, which first trains a CNN with roof labels and then makes roof pixel predictions, would easily introduce positioning errors for these buildings. Consequently, when performing building detection in urban areas with a large number of high-rises (e.g., in metropolises or most Chinese cities), the tilt effect of buildings would be a non-negligible issue related to localization precision. Although adopting true orthophotos instead of traditional ones can theoretically remove the residual tilt of buildings, the production of a true orthophoto relies heavily on a high-quality digital surface model (DSM) [7] or digital building model (DBM) [8]; however, the DSM could be unavailable in many situations (e.g., the DSM can hardly be derived when the imaging sensor is monocular) or may have limited quality, while the acquisition of DBM includes the building detection target itself to some extent. Therefore, in practice, images in the form of traditional orthophotos are still the main data source for building detection, especially from satellite images.

The reason for the above tilt effect is that the process of the traditional orthorectification only removes the terrain relief, while the ground objects, particularly the high-rises, are not rectified properly. As a result, in a traditional orthophoto, if we try to use the building roof outline to approximately represent its footprint, it might need to be further “rectified” by shifting to maximally align with the actual building footprint. Aiming at extracting such rectified footprints, in this approach, we propose a new feasible workflow for building footprint extraction from orthorectified aerial images. First, the roof label as well as the rectified footprint label are annotated for each building among the training images, and the facade label of the building is automatically derived. Then, a high-performance semantic segmentation CNN is trained and applied to test images, and the instance-level buildings with roof and facade segments are obtained by clustering the classified pixels. Finally, a simple optimization model is proposed to drive the extracted roof outline of every building to maximally align with its footprint; thus, the rectified footprint results can be generated.

We test the proposed workflow on a dataset containing aerial traditional orthophotos of a typical high-density residential area in Shanghai, which has 17,562 and 2371 buildings for training and test, respectively. As many other major cities in China, plenty of high-rises are within the test orthophotos, where obvious tilt effect can be observed. The experimental results show that the proposed workflow can extract obviously more accurate footprints than the baseline methods, which only use roof or footprint labels for training, and directly predicts segmentation results. The main findings or innovation points of our work are as follows:

- We observe a critical distinction between building roof and footprint extraction; by conducting comparative experiments, we verify that equating roof outline extraction to building mapping could lead to obvious errors.
- By classifying roof and facade pixels simultaneously, we propose a new workflow to extract rectified building footprint from traditional orthophotos, which can better adapt to the urban areas with a large amount of high-rises.
- We propose a simple optimization model for rectifying the locating deviation caused by the tilt effect, which can effectively improve the location accuracy for the identified buildings, especially for the high-rises.

The remainder of the paper is organized as follows. Section 2 provides a review for the related work. Section 3 introduces the dataset for experiment and presents the details for the proposed workflow and methods. Section 4 shows comparative experimental results. Section 5 makes further discussion about our workflow. Section 6 draws conclusions for the study.

## 2. Related Work

### 2.1. Building Segmentation

Before the popularity of deep learning, the general strategy of building segmentation from remote sensing images can be divided into two steps: (i) feature extraction, which performs explicit feature design and describes the image elements such as points, edges, or regions with spectral, textural, or other statistical information, and (ii) feature classification that labels the featured elements using classifiers such as random forest [9], adaptive boosting [10], and support vector machine [11]. The drawback of these methods is that the abstraction level of the extracted features is restricted by hand-crafted design, which would easily lead to limited generalization capability to heterogeneous scenes or imaging sensors.

The deep-learning-based methods, typified by CNN, have substantially reduced the dependence on manual feature design by adaptively learning high-dimensional features from images, thus significantly improving the performance of building segmentation [12,13]. The proposal of the fully convolution network (FCN) [14] is another milestone in semantic segmentation, which has been rapidly applied in building detection for its capability of directly and efficiently predicting full resolution segmentation results [15,16]. Later, many FCN-like networks with symmetric architectures such as U-Net [17], SegNet [18], and feature pyramid network [19] have become the mainstream deep-learning-based methods for building segmentation [20,21,22]; these networks are typically constructed by an encoder, which usually follows the design of the classic FCN, and a decoder, which upsamples the feature map and fuses multi-layer features. In order to enhance the performance of the architectures, further improvement, including feature selection [23] and multi-scale or multi-element feature fusion [24,25], have been proposed and applied to building segmentation. Currently, EfficientNet [26], which is developed by incorporating neural architecture search and scaling, has been proven as one of the state-of-the-art image recognition models and successfully applied to building detection [27].

Many open-source datasets [28,29] for building segmentation only provide footprints as ground truths. Learning from samples with footprint labels could be challenging for deep-learning models [30], because the training data may present two different and somewhat contradictory patterns [13]: for many low buildings, their footprints are mostly consistent with the roof outlines; for a high-rise, its footprint polygon may simultaneously partly include the roof and the facade (as shown in Figure 1). Therefore, other studies focus on roof segmentation and achieve high performance [4,5,6], but the segmentation results may still suffer from a lack of mapping accuracy due to the errors introduced by the misalignment between the roof and the footprint.

### 2.2. Misaligned Vector Correction and Relief Displacement

By far, few studies have developed specialized algorithms for automatically shifting the extracted roof outline to reduce its misalignment with the actual footprint. Similarly, there are several approaches that have tried to adaptively correct the displacement for the misaligned building vector data [31,32], the most typical of which is to improve the registration accuracy between open-source vector maps and remote sensing images [33]. However, guiding by the salient features on the roof boundary, these approaches can generally produce building vectors fitting well with roof outlines [34]. It means that, in traditional orthophotos, even if the building vectors have been updated after registration, they could still be displaced from the actual coordinates of the buildings, especially for the high-rises with obvious tilt effect.

The key point to address is that the influence brought by the tilt effect is compensating the offset deviation caused by the relief displacement. Relief displacement is a classic problem in photogrammetry which should be properly handled in many applications, such as image mosaicking [35], true orthophoto production [7], 3D building model constructing [36], and change detection [37]. Besides, the influence brought by relief displacement to building footprint extraction has also attracted attention. For example, Zhuo et al. segmented the image into roof, facade, and background pixels and successfully optimized the vector data from OpenStreetMap (OSM) to strictly align with the building footprint [38]. However, this approach is based on the existing OSM data; directly extracting building footprints from traditional orthophotos is still a rarely discussed problem.

### 2.3. Summary

In general, the characteristics of the proposed approach and the main relevant work can be summarized as in Table 1.

## 3. Materials and Methods

### 3.1. Data

**Figure 2:**
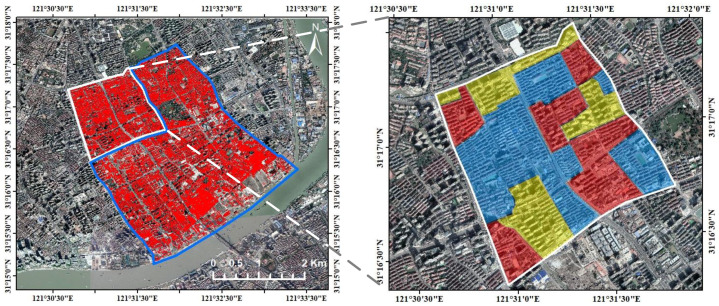
The study area in Waitan of Shanghai. The left image shows the split of the training and test areas, which are delineated by blue and white polygons, respectively; the red vectors represent the building instances within the study area. The right image shows the enlarged view for the test area, where the areas covering high-rise, mid-rise, and low-rise buildings are shaded in yellow, red, and blue, respectively.

As shown in Figure 2, a high-density residential area in Waitan of Shanghai is selected as the study region. We collected several aerial images captured by Leica DMC III airborne digital camera with 10 cm resolution for experiment. All the images are rectified into traditional orthophotos and merged as a mosaic. The whole study area covers about 11 km^2^ and is split into training and test areas, which include 8.9 km^2^, 17,562 buildings and 2.1 km^2^, 2371 buildings, respectively. The labels of the dataset are determined in a different way from other studies: for every building, the polygon encircling the roof outline is first annotated; then, if relief displacement (i.e., the tilt effect) can be observed for the building, the annotated polygon is duplicated and shifted to align with the footprint as accurately as possible. The two kinds of labels are both used for model training and accuracy evaluation. Besides, to explore the influence of relief displacement to detection accuracy of buildings with different heights, the test area is further divided and classified by visual checking into three categories: high-rise, mid-rise, and low-rise buildings (see Figure 2, right side).

### 3.2. Methods

**Figure 3:**
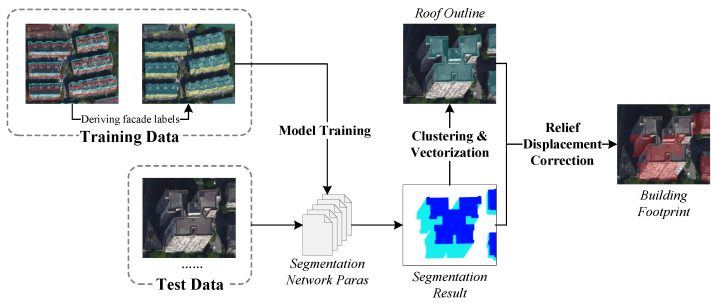
The proposed workflow for rectified building footprint extraction.

As shown in Figure 3, a new workflow is proposed in this approach for extracting rectified building footprints from traditional orthophotos. The workflow includes: (i) with the use of the annotated polygons of the roof and the rectified footprint, the facade label of every building is derived through several geoprocessing operations; (ii) the roof and facade labels are adopted for training a semantic segmentation network, which has a roughly symmetric design and employs the state-of-the-art version of EfficientNet as its backbone, in which the well trained segmentation network classifies the pixels of the input test image into roof/facade/background categories; (iii) the instance-level buildings with roof and facade segments are obtained by clustering the classified pixels, and the outline of the roof for each building is then extracted; and (iv) an energy function is constructed to drive the roof outline to align with the visible edges of the building footprint, and the final rectified footprint results can be generated.

#### 3.2.1. Training Label Preparation

**Figure 4:**
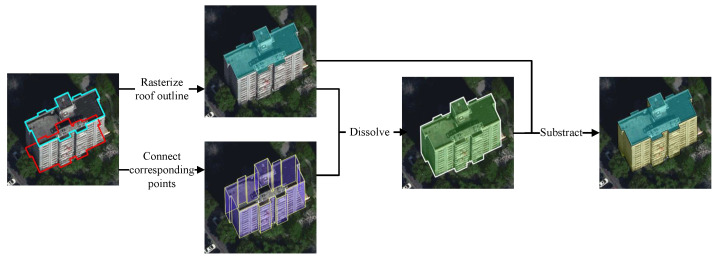
Derivation of the facade label with the roof and footprint polygons.

The key strategy of our approach is to simultaneously segment building roof and facade from the images. Thus, besides the labels of roof outlines, the facade labels also need to be prepared before model training. Directly annotating the facade label for a building could be time consuming. Instead, in our approach, several geoprocessing operations are applied to the roof and footprint labels for generating the facade label automatically. Figure 4 indicates the procedures of facade label generation. First, considering that the footprint polygon of every building is duplicated from the roof polygon, the one-by-one correspondence between vertices of the roof and footprint polygons can be easily determined. Since two adjacent vertices of the roof and the footprint can form a rectangle, which is a portion of the facade, the complete facade can be covered by constructing all the rectangles. Then, by dissolving the roof polygon along with all facade rectangles, the whole building containing the roof and facade, which we term as a complete building object (CBO), can be obtained. Afterwards, the facade label can be generated by subtracting the roof polygon from the CBO polygon.

#### 3.2.2. Segmentation for Building Roof and Facade

**Figure 5:**
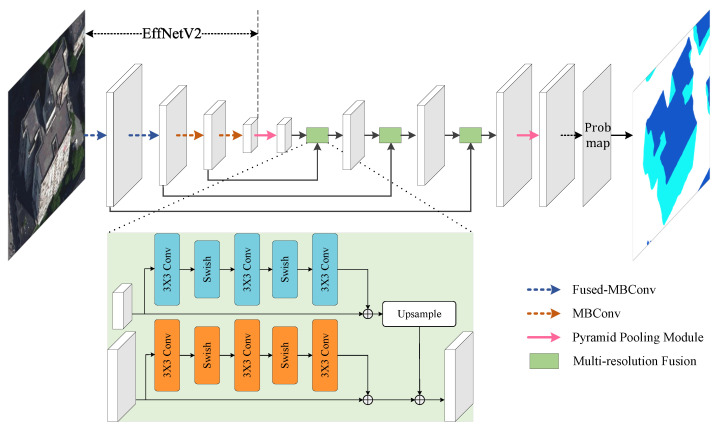
The architecture of the segmentation network designed for our approach.

Figure 5 shows the architecture of the framework designed for our building segmentation task. We use EfficientNetV2 [39] as the encoder of the structure due to its high performance on image recognition tasks. Following its original design, the block of mobile inverted bottleneck convolution (MBConv) is applied in the encoder for efficiently extracting fine-grained high-level features, while the MBConv blocks in the first fewer stages are replaced by the Fused-MBConv to further improve the efficiency. The features output by the encoder are gradually upsampled within the decoder, which produces the final features for building segmentation. Inspired by RefineNet [25], a multi-resolution fusion module is applied to the features of different scales for better capturing the contextual information. Specifically, in each stage of the decoder, the feature map with higher resolution in the encoder is reused along with the current feature map for generating new features. The two feature maps are both processed by the residual convolution blocks, each of which includes three 3×3 convolution kernels, with the first two followed by Swish activation functions [40]. The lower-resolution feature map after processing is upsampled and then merged with the higher-resolution one (i.e, element-wise addition) for generating the fusion result. Besides, we also employ the pyramid pooling module of the PSPNet [24], which is appended to the final feature map of the encoder as well as the decoder, to enhance the global information of the obtained features. Finally, an inference structure followed by a Softmax classifier is employed for predicting the probability map, which is then used for segmenting the input image pixels into roof/facade/background categories.

#### 3.2.3. Relief Displacement Correction

Following the operations conducted by [2], the probability map predicted by the segmentation network is post-processed to remove classification noises and the closed areas of the non-background pixels are clustered to generate CBO instances. In our approach, there are many CBO instances simultaneously including the roof as well as the facade segments; for these buildings, the position of their roof segments could be considerably offset from their actual coordinates due to relief displacement. Thus, a simple optimization model is proposed for adaptively translating the roof segment and maximally compensating the relief displacement. We define the energy function as $E=E_{area}+\alpha E_{boundary}$, where $E_{area}$ and $E_{boundary}$ represent two metrics, which jointly drive the roof segment toward the position of the building footprint; α is the weight parameter of $E_{boundary}$.

**Figure 6:**
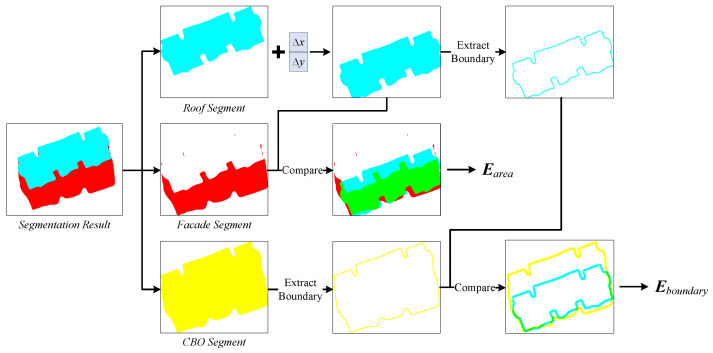
The computation of the metrics for defining the optimization energy function.

Figure 6 illustrates the computation procedures of the two metrics: (i) the segmentation result is first split into the roof segment and the facade segment, and the combination of the two is considered as the CBO segment; (ii) the roof segment is translated as a whole by adding a 2D correction vector (i.e., Δx and Δy), and the updated roof segment and the facade segment are compared to compute $E_{area}$; and (iii) the boundaries of the updated roof segment and the CBO are both extracted, and the buffer areas of the two boundaries are compared to compute $E_{boundary}$. We use the $E_{area}$ to impel the roof segment maximally overlapping with the facade, while the $E_{boundary}$ prevents the roof segment from crossing the CBO boundary.

The negative intersection over union (IoU) is used for representing the two energy items, which are defined as:

(1) $$\begin{array}{c} E_{area}=-\frac{A_{roof}\cap A_{facade}}{A_{roof}\cup A_{facade}} \end{array}$$

(2) $$\begin{array}{c} E_{boundary}=-\frac{B_{roof}\cap B_{CBO}}{B_{roof}\cup B_{CBO}} \end{array}$$

where $A_{roof}$ and $A_{facade}$ represent the areas of the roof segment and the facade segment, respectively; $B_{roof}$ and $B_{CBO}$ represent the boundary buffer areas of the roof segment and the CBO segment, respectively.

The target of the above optimization problem is to find the Δx and Δy that minimize *E*. Searching the whole possible range pixel by pixel could be time consuming; thus, a simple coarse-to-fine searching strategy is used to find the optimal position. First, since the updated roof segment is supposed to locate within the CBO, the whole searching space $S_{H,W}$ can be easily determined; then, $S_{H,W}$ is divided into 10×10 sectors, in which the sector with its center point minimizing *E* can be selected as the most promising object area for more refined search; and, finally, the optimal position can be determined by performing pixel-wise searching within the object area.

## 4. Results

### 4.1. Implementation Details

We implement our algorithms in Python on a 64-bit Ubuntu system. The segmentation network is trained and tested in PyTorch [41] with 6 NVIDIA GeForce RTX 2080Ti GPUs. The encoder of the network follows the configuration of EfficientNetV2-S [39] due to the limited volume of the training data. We set the crop size as 768×768, the batch size as 12, the initial learning rate as 0.001, and train over the whole training set for 100 epochs, during which the learning rate is decreased by half at the 30th, 50th, and 80th epoch. The optimizer of stochastic gradient descent with a weight decay of 0.0001 and a momentum of 0.9 is employed for model training.

The buffer size for comparing boundaries of the roof and the CBO is set as 3 pixels. The weight parameter α in the proposed energy function is set as 2.25 after tuning. Considering the segmentation error, the CBO’s bounding box plus 30 pixels of padding is considered as the moving border of the roof segment to determine $S_{H,W}$. The traced boundary of the roof segment is simplified by applying the Douglas–Peucker algorithm [42] with threshold of 3 pixels, which helps generate the final vectorized footprint for a building after relief displacement correction.

### 4.2. Baselines and Evaluation Metrics

The primary motivation of this approach is to validate the necessity and feasibility of the proposed new workflow. Therefore, we use two representative building segmentation workflows as the baseline methods for comparison:

- Baseline-1. For this workflow, the segmentation network is trained with labels that delineate the roof outlines and the segmentation target is focused on the rooftop [5,6].
- Baseline-2. The network is trained with footprint labels, which could be obviously misaligned with the roof outlines for high-rise buildings. The prediction of this workflow is expected to represent the location of the building footprint [30]. Most studies that directly use a SpaceNet [28] or INRIA [29] dataset can be classified as this workflow.

The two baselines share the same algorithms with the proposed approach, but the main difference is the input training labels of the workflow: the baselines use only single types of labels for model training and conduct binary segmentation, while our approach takes relief displacement into consideration and simultaneously generates results of roof outlines and footprints.

Following related studies [13,43], the metrics of IoU [44], F1-score, Precision, and Recall are used for evaluating the segmentation accuracy. For our approach, the prediction results of the roof outlines and the footprints are separately evaluated by the human-annotated roof and footprint labels, respectively. For the two baselines, to explore how much the roof predictions can be used as footprints (or vice versa), the results are evaluated by not only the roof but also the footprint labels.

### 4.3. Overall Comparison

Table 2 shows the quantitative comparison results of the three workflows for the whole test set. Generally, the proposed workflow achieves obvious improvement over the two baselines in terms of both roof outline results and footprint results. Our improved workflow generates building footprints with higher accuracy than Baseline-2 (0.794 vs. 0.720 in IoU), which demonstrates that directly training the segmentation network with footprint labels can lead to certain accuracy loss. Our workflow also achieves higher roof segmentation accuracy than Baseline-1 (0.883 vs. 0.856 in IoU) by gaining a 3.2% increment of Recall, revealing that the additional facade labels can help the network better understand the semantic pattern of the rooftop. Another important finding is that, although Baseline-1 presents good performance in roof segmentation, the accuracy of its results decreases sharply (from 0.856 to 0.626 in IoU) when evaluated by footprint labels, which verifies that for areas with high-rise buildings, performing deep-learning-based building mapping by equating the roof outline to its footprint position could lead to significant errors. Meanwhile, the results of Baseline-2 achieve poor accuracy whether evaluated by the footprint labels or the roof labels, indicating that the model could be misguided by the labels including both the roof and the facade textures.

**Figure 7:**
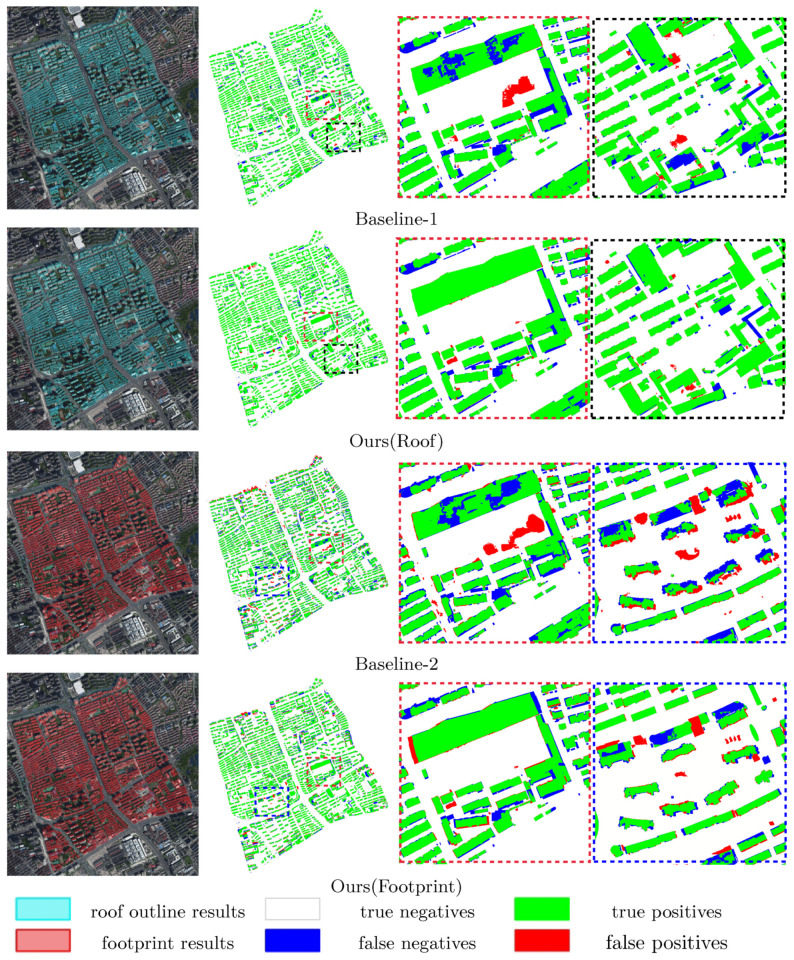
Evaluation of the three workflows for the overall test area. The results of Baseline-1 and Baseline-2 are polygons shaded in cyan and red on the images; the two workflows are visually evaluated by the roof labels and the footprint labels, respectively. Our workflow simultaneously generates roof outlines and footprints, which are separately evaluated by the roof labels and the footprint labels, respectively.

Figure 7 presents the overall prediction results and their evaluation maps of the three workflows. As for the roof predictions, our workflow exhibits higher capability of extracting complete roof segments than Baseline-1 (e.g., the large buildings within the red rectangles). Besides, the road area covered by crowded cars is much more easily mistaken as roof area by Baseline-1 than our workflow (i.e., areas within the black rectangles), indicating that the proposed workflow leads to better semantic understanding of building rooftops. As for the footprint predictions, Baseline-2 omits considerable parts of the large buildings’ footprints within the red rectangles, while our workflow is able to extract relatively complete results. The blue rectangles point out several high-rise buildings with large relief displacement, where our workflow obviously outperforms Baseline-2 by well suppressing the false detection.

**Figure 8:**
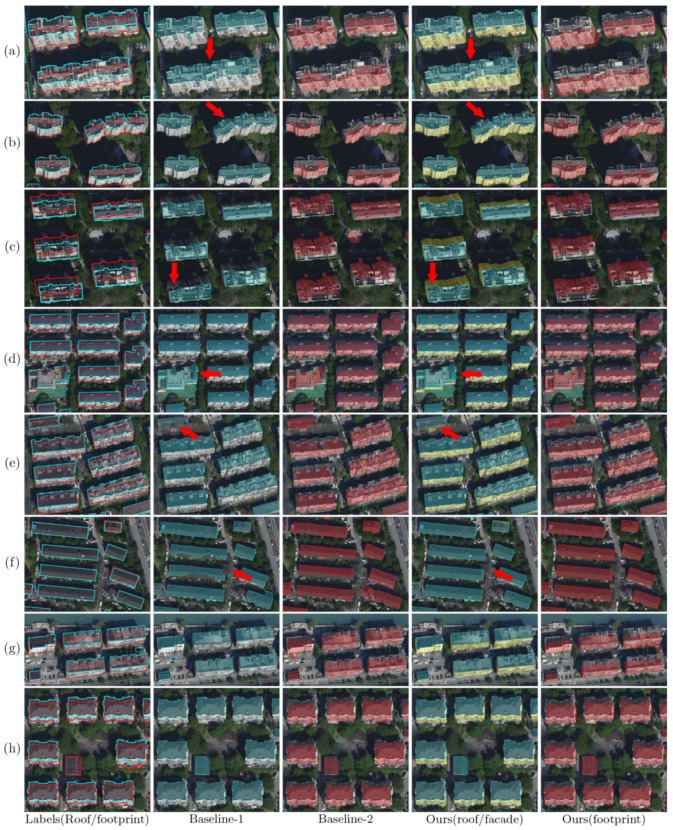
Results of the three workflows in typical scenarios. (**a**–**c**): high-rise buildings; (**d**,**e**): middle-rise buildings; (**f**–**h**): low buildings. The leftmost column shows the annotated roof and footprint labels of the test images, which are delineated by cyan and red polygons, respectively. The roof and footprint predictions of the three workflows are shaded in cyan and red on the images, respectively. The facade predictions of our workflow are also presented (in the fourth column, shaded in yellow).

As shown in Figure 8, typical scenarios are selected from the test set for making further comparison between the three workflows. Generally, Baseline-2 can identify the buildings at object-level, but often fails in accurately extracting the geometric shape of the footprints. In comparison, our workflow makes good use of the edge information from the roof outlines, thus generating more accurate and geometrically reasonable footprint results. On the other hand, the results demonstrate that our workflow outperforms Baseline-1 by better capturing the details of the roof outlines. As pointed out by the red arrows, our workflow is more robust to the shadow cast by the high-rises (i.e., Figure 8a–c), the building with complicated geometric roof shape (i.e., Figure 8d), and the background with similar color to the rooftop (i.e., Figure 8e,f).

## 5. Discussion

### 5.1. The Advantage of the Proposed Approach

The impact of the relief displacement to extracting and positioning buildings from traditional orthophotos is adequately considered in our approach. We improve the typical workflow of building segmentation by using additional facade labels and applying relief displacement correction to the extracted roof segments. The experimental results demonstrate that the proposed workflow obviously outperforms the typical workflows (i.e., the two baselines) in high-density residential areas. The workflow that focuses on roof segmentation [5,6] can generally extract high-quality roof outlines, but those outlines can hardly represent the position of the building footprints accurately. Directly training the segmentation network with footprint labels [30] is also proven to cause loss of accuracy in our experiment, most likely because the complex pattern of the building footprint in the traditional orthophoto, to some extent, challenges the learning capability of the model.

Different from other studies, the proposed workflow requires two annotations (i.e., the roof polygon and the rectified footprint polygon) for a single building when performing model training. However, the additional time cost for sample preparation is not significant, since the only added operation after delineating the roof outline is duplicating the polygon and translating it to align with the footprint; for buildings that present no relief displacement, no additional operation is required for label annotation. Therefore, we believe that the additional cost of our workflow is within an acceptable level in contrast to the achieved accuracy improvement, especially for areas densely covered by high-rise buildings.

### 5.2. The Effectiveness of the Design Options

Currently, training the segmentation network with polygon labels and making binary pixel-wise classification is the mainstream workflow for building detection from remote sensing images. The additional designs of our approach over traditional solutions are two-fold: (i) three-category segmentation with roof and footprint labels and (ii) the module of relief displacement correction. When ablating the displacement correction module, our workflow can only generate roof outline predictions; using the roof outline results for representing building footprints leads to a much larger error (0.643 vs. 0.794 in IoU), which demonstrates the necessity of correcting the relief displacement in our approach. As for the segmentation module, the proposed workflow degrades to Baseline-1 if not using additional facade labels for model training; the evaluation results in Table 2 has proven that importing facade labels helps producing finer roof segmentation results.

In terms of time-consuming, after training, the proposed framework generates the three-category segmentation results for the whole test area (18,567×20,568 pixels in total) in 818.9 s via a GPU and finishes the process of displacement correction for 1190 buildings within the test area in 1411.8 s via a single CPU. The efficiency of the proposed workflow should be acceptable for most general applications.

### 5.3. Detection Accuracy of Areas with Different Building Height

As listed in Table 3, the results of the test areas with high-rise, mid-rise, and low-rise buildings are evaluated separately for further analysis. The evaluation shows that the advantage of our workflow is considerably magnified for the high-rise buildings. When evaluated by the footprint labels, the improvement of our workflow over Baseline-2 increases to 10.8%, while Baseline-1 presents a significant worse overall accuracy (0.391 vs. 0.732 in IoU), which fully demonstrates the necessity of correcting the relief displacement within these areas. In general, the difference between our workflow and the baselines gradually narrows from high-rise buildings to low-rises, but even for low buildings, our workflow still gains 7.5% increment of IoU over Baseline-2, indicating that the performance of the model trained solely by the footprint labels could be weakened by the samples with large relief displacement. The difference between the proposed workflow and Baseline-1 is relatively small when evaluating by the roof labels, especially for the low buildings, which means that adopting our workflow could be unnecessary if relief displacement is rarely observed in the application scenarios.

**Figure 9:**
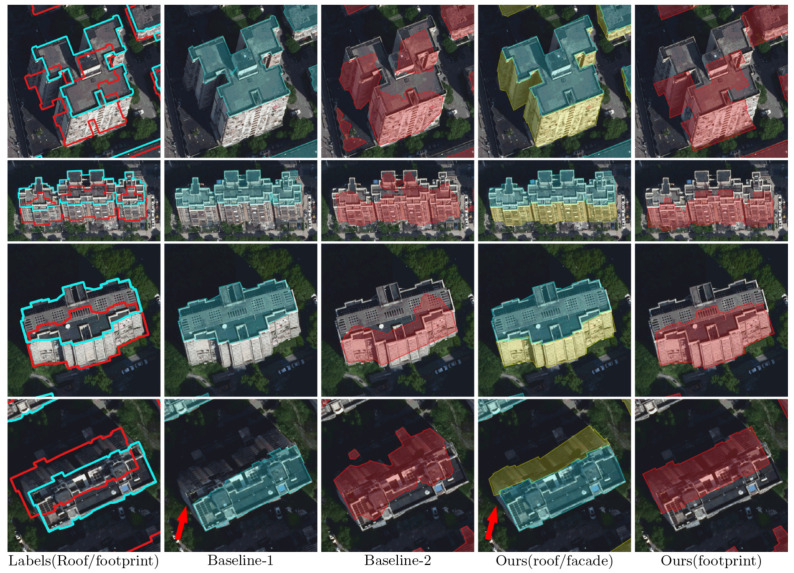
Results of the three workflows for typical high-rise buildings. The leftmost column shows the annotated roof and footprint labels of the test images, which are delineated by cyan and red polygons, respectively. The roof and footprint predictions of the three workflows are shaded in cyan and red on the images, respectively. The facade predictions of our workflow are also presented (in the fourth column, shaded in yellow).

**Figure 10:**
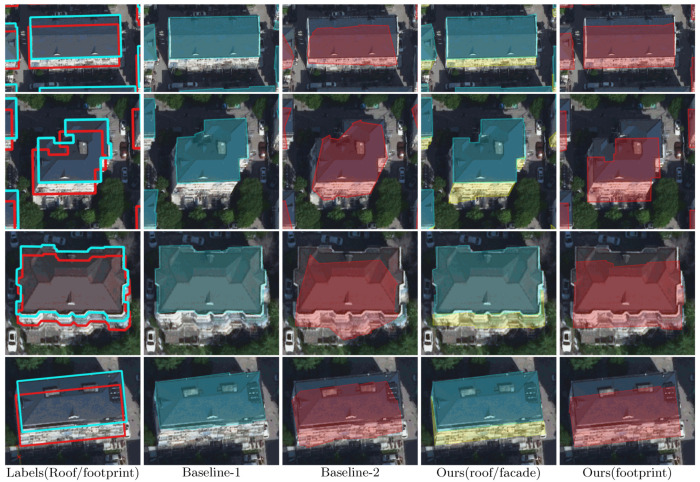
Results of the three workflows for typical mid-rise buildings. The leftmost column shows the annotated roof and footprint labels of the test images, which are delineated by cyan and red polygons, respectively. The roof and footprint predictions of the three workflows are shaded in cyan and red on the images, respectively. The facade predictions of our workflow are also presented (in the fourth column, shaded in yellow).

**Figure 11:**
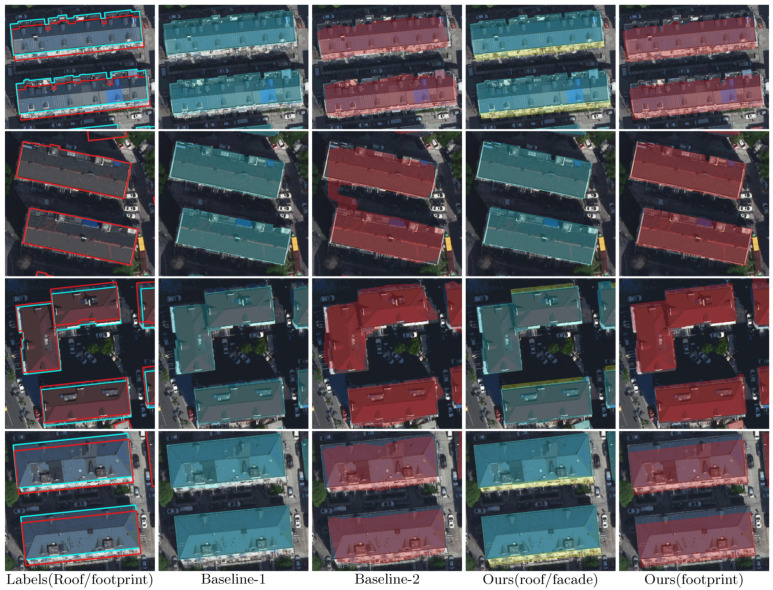
Results of the three workflows for typical low-rise buildings. The leftmost column shows the annotated roof and footprint labels of the test images, which are delineated by cyan and red polygons, respectively. The roof and footprint predictions of the three workflows are shaded in cyan and red on the images, respectively. The facade predictions of our workflow are also presented (in the fourth column, shaded in yellow).

Figure 9, Figure 10 and Figure 11 illustrate the results of the three workflows for typical high-rise, mid-rise, and low-rise buildings, respectively. For the high-rises, it can be seen from Figure 9 that the buildings have significant tilt effect, which largely challenges the recognition performance of Baseline-2, leading to geometrically irregular and sometimes fragmented segmentation results. Meanwhile, our workflow slightly outperforms Baseline-1 by recovering more complete roof boundaries (as pointed by the red arrows), demonstrating the advantage of applying additional facade labels in building segmentation. Similar conclusions can be made for the mid-rise buildings in Figure 10; although the tilt effect is relatively unnoticeable, Baseline-2 still fails in extracting regular footprint polygons. For the low-rise buildings, Figure 11 shows that our workflow can accurately correct the relief displacement even if the imaging area of the building facades is quite small. In comparison, Baseline-1 achieves a similar performance with ours in roof segmentation, but Baseline-2 still seems not to have learned a stable pattern for footprint extraction.

## 6. Conclusions

In this study, an improved workflow is proposed for extracting more accurate building footprints from traditional orthophotos of aerial images. Different from previous studies, the tilt effect of buildings is fully considered in our workflow. A deep-learning-based segmentation network is constructed for obtaining roof and facade segments from images; additionally, a module of relief displacement correction is applied for compensating the locating deviation of the extracted roof segments. The experiments on a dataset of a high-density residential area in Shanghai demonstrate that our workflow can generate more accurate results of roof outlines (with IoU of 0.883) and building footprints (with IoU of 0.794) than the baseline methods. The comparative analysis verifies that the first baseline, which conducts strict rooftop segmentation, generates high-quality roof extraction results, but those results can hardly be equated to accurate building footprints. The second baseline, which trained the segmentation network solely with footprint labels, generally fails to produce building footprints with high accuracy or regular geometry, especially for the high-rise buildings. Nevertheless, the proposed workflow also has limitations. For example, a multi-layer structured building may have two or more blocks with varying degrees of relief displacement. This is currently challenging for us since our workflow would consider the multiple building blocks as one complete object. The subsequent study will take this problem into consideration. Besides, the applicability of the proposed workflow to satellite orthophotos will also be explored in the future. 

## Figures and Tables

**Table 1:** Comparison of the characteristics between previous work and ours.

Approaches	Training Labels		Displacement Correction	Pros and Cons
	Roof	Footprint		
[5,6]	√			The learning task is clearly defined as roof segmentation, but errors could be introduced by relief displacement.
[28,29,30]		√		The collected footprint labels can be used without further refinement, but the patterns of the training data are challenging for learning.
[33]	√		√	Registration of collected labels is conducted guiding by roof boundary features, but displacement may remain due to obvious tilt effect.
Ours	√	√	√	Relief displacement is fully considered by performing 3-category segmentation, but additional labels are required for model training.

**Table 2:** Evaluation results of the three workflows for the whole test set. For every metric, the highest value is highlighted in bold.

Workflows	Reference: Roof				Reference: Footprint			
	IoU	F1-Score	Precision	Recall	IoU	F1-Score	Precision	Recall
Baseline-1	0.856	0.922	**0.961**	0.886	0.626	0.770	0.801	0.741
Baseline-2	0.613	0.760	0.798	0.726	0.720	0.837	0.878	0.800
Ours	**0.883**	**0.938**	0.958	**0.918**	**0.794**	**0.885**	**0.904**	**0.866**

**Table 3:** Evaluation results of the three workflows for areas with different building height. For every metric, the highest value is highlighted in bold.

Building Type	Workflows	Reference: Roof				Reference: Footprint			
		IoU	F1-Score	Precision	Recall	IoU	F1-Score	Precision	Recall
High-rise	Baseline-1	0.833	0.909	**0.966**	0.859	0.391	0.581	0.543	0.562
	Baseline-2	0.465	0.635	0.618	0.653	0.624	0.800	0.740	0.769
	Ours	**0.864**	**0.927**	0.951	**0.904**	**0.732**	**0.865**	**0.826**	**0.845**
Mid-rise	Baseline-1	0.816	0.899	**0.960**	0.845	0.622	0.798	0.738	0.767
	Baseline-2	0.609	0.757	0.778	0.737	0.690	0.886	0.758	0.817
	Ours	**0.860**	**0.925**	0.959	**0.893**	**0.766**	**0.899**	**0.838**	**0.867**
Low-rise	Baseline-1	0.883	0.938	**0.963**	0.914	0.754	0.868	0.852	0.860
	Baseline-2	0.714	0.833	0.848	0.818	0.756	0.915	0.813	0.861
	Ours	**0.900**	**0.947**	0.959	**0.936**	**0.831**	**0.919**	**0.897**	**0.908**

## Data Availability

Data sharing is not applicable to this article.
